# 2-Deoxy-D-glucose and combined 2-Deoxy-D-glucose/albendazole exhibit therapeutic efficacy against *Echinococcus granulosus *protoscoleces and experimental alveolar echinococcosis

**DOI:** 10.1371/journal.pntd.0010618

**Published:** 2022-07-18

**Authors:** Qi Xin, Wei Lv, Yunxi Xu, Yumei Luo, Caifang Zhao, Bichen Wang, Miaomiao Yuan, Huanping Li, Xiaoxia Song, Tao Jing

**Affiliations:** 1 Department of Pathogenic Biology, School of Basic Medical Sciences, Lanzhou University, Lanzhou, China; 2 The Eighth Affiliated Hospital, Sun Yat-sen University, Shenzhen, China; University of Würzburg, GERMANY

## Abstract

2-Deoxy-D-glucose (2-DG) is a glucose analog used as a promising anticancer agent. It exerts its effects by inhibiting the glycolytic energy metabolism to deplete cells of energy. The larval stage of *Echinococcus* relies on glycolysis for energy production. Therefore, in this study, we investigated the *in vitro* and *in vivo* efficacy of 2-DG against the larval stage of *Echinococcus granulosus* and *E*. *multilocularis*. 2-DG exhibited significant time- and dose-dependent effects against *in vitro* cultured *E*. *granulosus* protoscoleces and *E*. *multilocularis* metacestodes. A daily oral administration of 500 mg/kg 2-DG in *E*. *multilocularis*-infected mice effectively reduced the weight of metacestodes. Notably, the combination treatment, either 2-DG (500 mg/kg/day) + albendazole (ABZ) (200 mg/kg/day) or 2-DG (500 mg/kg/day) + half-dose of ABZ (100 mg/kg/day), exhibited a potent therapeutic effect against *E*. *multilocularis*, significantly promoting the reduction of metacestodes weight compared with the administration of 2-DG or ABZ alone. Furthermore, the combination significantly promoted apoptosis of the cells of metacestodes and inhibited glycolysis in metacestodes, compared with the administration of 2-DG or ABZ alone. In conclusion, 2-DG exerts an effective activity against the larval stage of *Echinococcus*. Thus, it may be a promising anti-*Echinococcus* drug, and its combination with ABZ may provide a new strategy for the treatment of echinococcosis in humans.

## Author summary

Echinococcosis is a serious but neglected helminthic zoonosis caused by the larval stage of *Echinococcus granulosus* and *E*. *multilocularis*. At present, clinical pharmacotherapy of echinococcosis, such as albendazole (ABZ) and mebendazole, has limited effectiveness. Thus, the development of novel therapeutic drugs for human echinococcosis is urgently needed. 2-Deoxy-D-glucose (2-DG) is a glucose analog used as a promising anticancer agent, and it exerts its effects by inhibiting the glycolytic energy metabolism to deplete cells of energy. *Echinococcus* in the host depends on glycolysis for energy production and glycolysis intermediates for other metabolic processes. Therefore, in this study, we investigated the efficacy of 2-DG against *Echinococcus*. 2-DG exerted an effective *in vitro* and *in vivo* activity against *E*. *granulosus* protoscoleces and *E*. *multilocularis* metacestodes, and the combination of this drug with ABZ further improved the therapeutic effect. Therefore, 2-DG can be developed as a promising anti-*Echinococcus* drug, and its combination with ABZ may provide a new strategy for the treatment of human echinococcosis in the future.

## Introduction

Echinococcosis is a cosmopolitan zoonosis caused by the larval stage of the genus *Echinococcus*. Humans are mainly infected as an intermediate host by two types of echinococcosis: cystic echinococcosis (CE) and alveolar echinococcosis (AE). CE caused by *Echinococcus granulosus* is distributed worldwide, it mainly occurs in some regions of South America, Asia, Western and Central Europe, Australia, and North Africa [1]. Carnivorous animals (usually dogs) are the definitive host of *E*. *granulosus*. AE caused by *E*. *multilocularis* is generally endemic in the northern hemisphere, with the highest prevalence in Northern and Eastern Europe, Japan, Canada, and China (particularly in areas of Xinjiang, Qinghai, and Ningxia) [2]. Carnivores, mainly foxes, dogs, and cats, are the definitive host of *E*. *multilocularis*. Adult cestodes of both *E*. *granulosus* and *E*. *multilocularis* live within the small intestine of their host, and they produce and release eggs into the environment with feces. Humans can accidentally acquire the infection by ingesting eggs shed by the definitive hosts. Upon infection, metacestodes proliferate asexually in the organs (primary the liver), thereby causing space-occupying lesions, organ malfunction, and even death [3].

The treatments for CE include surgery, PAIR (Puncture, Aspiration, Injection, Reaspiration), and chemotherapy. For AE, the major treatment option is surgical resection complemented by pre- and postoperative anti-parasitic treatment [4]. However, in patients without specific symptoms that would require surgical therapy, chemotherapy remains an effective option. The drugs presently used for the treatment of echinococcosis are the benzimidazole-derivatives (BMZs) mebendazole and albendazole (ABZ). However, both drugs have a limited cure rate (30%) in the treatment of CE. Previous studies have shown that BMZs have increased the 10-year survival rate of inoperable or non-radically operated AE patients from 6%–25% to 80%–83% [5, 6]; however, given that BMZs do not exert a parasitocidal but parasitostatic effect *in vivo*, many AE patients have to undergo treatment for extended periods of time, often lifelong. Therefore, considering the unsatisfactory results of the treatment with BMZs, as well as the elevated risk of adverse effects during treatment [5], it is necessary to find novel therapeutic drugs for more effective treatment of echinococcosis.

In recent years, a number of compounds with potential effects against *Echinococcus* metacestodes have been investigated *in vitro* and/or in mice models [7]. These compounds, including nitazoxanide [8], imatinib [9], artemisinin and artemisinin derivatives [10], tamoxifen [11], bortezomib [12], 5-fluorouracil and paclitaxel [13, 14], menthol [15], atovaquone [16], and metformin [17, 18], have demonstrated notable effects against *Echinococcus*. In our previous study, we have demonstrated the effectiveness of a series of glycolytic inhibitors against both *E*. *granulosus* and *E*. *multilocularis* [19, 20]. Among the inhibitors of hexokinase, 3-bromopyruvate has shown the highest anti-*Echinococcus* activity, which suggests that the inhibition of glycolysis in metacestodes mediated by hexokinase may be an effective anti-parasitic strategy against *Echinococcus*. 2-Deoxy-D-glucose (2-DG), a non-metabolized glucose analog and an inhibitor of glucose transport and glycolytic energy metabolism, is the most widely investigated metabolic inhibitor for targeting glucose metabolism [21]. 2-DG has been proven as an antiviral drug [22] and a promising treatment agent for many cancers [23–25]. Furthermore, 2-DG has displayed antiplasmodial activity [26], anti-cryptosporidial activity [27, 28], and inhibition of *Besnoitia besnoiti* replication [29]. However, there is hitherto no evidence of the effect of 2-DG on echinococcosis. Therefore, in this study, we assessed the potential therapeutic implications of 2-DG on *Echinococcus* metacestodes by investigating the *in vitro* and *in vivo* effects of 2-DG against *E*. *granulosus* and *E*. *multilocularis*. We also analyzed the effect of 2-DG on apoptosis and glucose metabolism of metacestodes cells to explore the possible underlying mechanisms.

## Methods

### Ethics statement

Animal procedures and management were conducted in compliance with the protocols (jcyxy20200401) approved by the Institutional Animal Caring and Using Committee of Lanzhou University. The mice were maintained in laboratory conditions with 12 h light/dark cycle and controlled temperature, and they had free access to water and commercial mouse chow throughout the study.

### Chemicals

ABZ, nitazoxanide (NTZ), 2-DG, and 4’,6-diamidino-2-phenylindole (DAPI) were obtained from Sigma-Aldrich (St. Louis, MO, USA). All tissue culture media and fetal calf serum (FCS) were purchased from Hyclone (Logan, UT, USA).

### *In vitro* drug activity study on *E*. *granulosus* protoscoleces

*E*. *granulosus* protoscoleces were isolated from hydatid cysts in the liver of naturally infected sheep slaughtered in an abattoir (Xining, Qinghai province, China) by aseptic aspiration with a hypodermic syringe (20 mL, 18 gauge needle). The *in vitro* drug treatment of protoscoleces was performed as previously described [30]. Briefly, the collected protoscoleces were washed twice in Hanks’ balanced salt solution, transferred to 25-cm^2^ cell culture flask containing RPMI 1640 culture medium (supplemented with 12 mM HEPES, 2 mM glutamine, 100 U/mL penicillin, 100 μg/mL streptomycin, and 10% FCS), and incubated in an incubator at 37°C and 5% CO_2_ conditions. The protoscoleces were cultured 3 days for further *in vitro* drug treatment. The treatments were performed in 24-well tissue culture plates containing 100 viable and morphologically intact protoscoleces per well and 1 mL of culture medium without both FCS and phenol red. 2-DG and NTZ were dissolved in dimethyl sulfoxide (DMSO) and added to the wells. 2-DG at a serial concentration of 10, 20, 40, 80, 160, and 320 μM was used for the experiment, and NTZ (40 μM) served as a positive control. In all drug conditions, the final concentration of DMSO was 0.2%. Protoscoleces incubated in the culture medium containing 0.2% DMSO were used as negative controls. The mortality of the protoscoleces was assessed daily by the trypan blue staining test and observed on an inverted microscope. Each drug concentration was performed in duplicate, and the experiments were repeated twice. Additionally, ultrastructural study with scanning electron microscopy (SEM) was performed.

### *In vitro* drug activity study on *E*. *multilocularis* metacestodes

*In vitro* cultivation of *E*. *multilocularis* metacestodes (isolate Xinjiang) was performed as previously described, with few modifications [31]. In short, *E*. *multilocularis* metacestodes were isolated aseptically from Mongolian gerbils (*Meriones unguiculatus*) infected via intraperitoneal injection of minced metacestodes. Then, metacestodes were cut into tissue blocks of 0.5 cm^3^ and washed twice in Hanks’ balanced salt solution. Three pieces of tissue were placed in a 25-cm^2^ cell culture flask that had been precultured with human liver SMMC-7721 cells, containing Dulbecco’s modified Eagle medium [DMEM], supplemented with 12 mM HEPES, 2 mM glutamine, 100 U/mL penicillin, 100 μg/mL streptomycin, and 10% FCS. These co-cultures were incubated at 37°C and 5% CO_2_, with changing the medium thrice a week. After 1 to 2 months of culture, metacestode vesicles that reached 2–4 mm in diameter were harvested from the medium for *in vitro* drug treatment. After being washed three times in Hanks’ balanced salt solution, the vesicles were distributed to 24-well plates (Corning Inc., New York, NY, USA) with approximately 30 vesicles in 2 mL of RPMI 1640 culture medium without both FCS and phenol red (supplemented with 2 mM glutamine, 100 U/mL penicillin, 100 μg/mL streptomycin) per well. 2-DG at serial concentrations of 10, 20, 40, 80, 160, and 320 μM was used for the treatment, and NTZ (40 μM) served as a positive control. Metacestodes vesicles incubated in a culture medium containing 0.2% DMSO were used as negative controls. After 36 and 120 hours of incubation, 300 μL of the culture medium from all of the groups was harvested and centrifuged at 10,000*g* for 30 minutes at 4°C, and then the supernatants were stored at −20°C for *E*. *multilocularis* alkaline phosphatase (*Em*AP) activity assays. Each drug concentration was performed in duplicate, and experiments were repeated twice.

The quantitative assessment of *Em*AP activity was performed as previously described [32]. In short, 30 μL of the supernatant was mixed with 170 μL of alkaline phosphatase substrate buffer (0.5 M ethanolamine, 0.5 mM MgCl_2_ [pH 9.8]) containing *p*-nitrophenyl phosphate (1 mg/mL) in 96-well microtiter plates (Corning Inc., New York, NY, USA). Then, the plates were incubated at 37°C for 30 minutes, and finally, *A*_405_ values were read on an enzyme-linked immunosorbent assay (ELISA) reader (Bio-Tek, Winooski, VT, USA). The experiments were performed in triplicates.

### *In vivo* efficacy study in mice infected with *E*. *multilocularis* metacestode

Eight-week-old female BALB/c mice were infected by intraperitoneal inoculation with homogenized *E*. *multilocularis* metacestodes (isolate Xinjiang). Eight weeks after the inoculation, the mice were randomly divided into the following five groups (n = 7 per group): (1) Untreated control group, which received 200 μL honey/0.5% carboxymethyl cellulose (CMC) (1:1 v/v); (2) ABZ group, which received 200 mg/kg/day ABZ suspension; (3) 2-DG group, which received 500 mg/kg/day 2-DG suspension; (4) ABZ + 2-DG group, which received a combination of 200 mg/kg/day ABZ and 500 mg/kg/day 2-DG suspension; (5) ABZ half-dose + 2-DG group, which received a combination of 100 mg/kg/day ABZ and 500 mg/kg/day 2-DG suspension. All drugs were suspended in honey/0.5% CMC (1:1 v/v) and were applied orally in a volume of 200 μL/mouse. The treatments were performed daily during a period of 6 weeks. At the end of the study, the mice were sacrificed, and necropsy was performed. The metacestode cysts were isolated from the peritoneal cavity of the mice, and the weight of the cysts was recorded for efficacy assessment.

### Blood biochemical parameters and cytokines assay

At the end of the study, blood samples from ocular sinus were collected from the mice, and serum was separated by centrifugation at 3,000*g* for 10 minutes. The serum parameters, including total protein (TP), albumin (ALB), globulin (GLB), alanine aminotransferase (ALT), aspartate aminotransferase (AST), total bilirubin (TBIL), alkaline phosphatase (ALP), glutamyltranspeptidase (GGT), creatinine (CREA), and urea, were measured on the ROCHE cobas 8000 biochemical analyzer (ROCHE, Basel, Switzerland). The serum levels of IFN-γ and IL-10 were determined using mouse ELISA kits IFN-γ and IL-10 (Dakewe Biotech Company Ltd., Shenzhen, China), respectively. The absorbance was read at 450 nm. Each assay was performed in duplicate.

### Morphological investigations

Metacestodes isolated from the mice involved in the efficacy study were fixed in 2.5% glutaraldehyde in 100 mM sodium cacodylate buffer (pH 7.2) for the SEM and transmission electron microscopy (TEM) investigations as previously described [20]. The samples were observed and photographed with a Hitachi S-450 SEM and a JEOL JEM-1230 TEM, respectively. Meanwhile, the metacestode cysts, livers, and spleens of the mice were fixed in 10% of formaldehyde, embedded in paraffin, sectioned, and stained with hematoxylin and eosin (H&E) for histopathological examination.

### Assay for apoptosis in metacestodes of *E*. *multilocularis*

Apoptotic cells in the cysts were identified using the terminal deoxynucleotidyl transferase-mediated dUTP nick end labeling (TUNEL) method. The assay was performed using a TUNEL kit (Beyotime Institute of Biotechnology, Jiangsu, China) in accordance with the manufacturer’s instructions. Briefly, the formaldehyde-fixed paraffin-embedded metacestode cysts, which were recovered from the mice involved in the efficacy study, were sliced into 5-μm-thick sections. Then, after deparaffinization in xylene and dehydration in an ethanol series, the sections were treated with 20 μg/mL proteinase K at 25°C for 20 minutes. Subsequently, they were washed three times with PBS. After incubation with equilibration buffer at 25°C for 30 minutes, they were exposed to terminal deoxynucleotidyl transferase (TdT) incubation buffer consisting of FITC-12-dUTP and recombinant TdT enzyme at 37°C for 60 minutes. After washing three times with PBS containing 0.1% Triton and 5 mg/mL bovine serum albumin, the sections were counterstained with DAPI by incubation with 2 μg/mL DAPI buffer at 25°C for 5 minutes. The negative control was exposed to labeled solution consisting of FITC-12-dUTP but without recombinant TdT enzyme. Finally, 20×images were captured with a fluorescence microscope (BX53, Olympus, Tokyo, Japan) at a constant exposure time. The integrated density of green fluorescence in metacestodes was analyzed on the 20×images using ImageJ software. The threshold was conducted at a constant value (Default, Red) for all images.

### Assay for glucose consumption and lactic acid production

Freshly excised *E*. *multilocularis* metacestodes from the infected Mongolian gerbils were washed three times at 4°C in Chance Hess Medium (6.2 mM KCl, 154 mM NaCl, and 11 mM NaPi, pH 7.4). The metacestodes were cut into slices (1-mm-thick). Then, 200 μg each was incubated for 30 minutes at 37°C in 2 mL of Chance Hess Medium containing various concentrations of ABZ (20 μM, 80 μM, 320 μM) and 2-DG (12.5 mM, 50 mM, 100 mM, 200 mM). In order to investigate the drug combination on glucose metabolism of metacestodes and the effect of ABZ + 2-DG and ABZ half-dose + 2-DG, metacestodes of the same weight were incubated with various concentrations of ABZ + 2-DG (10 μM + 12.5 mM, 20 μM + 12.5 mM, 40 μM + 50 mM, 80 μM + 50 mM, 160 μM + 200 mM, 320 μM + 200 mM) under same conditions. Due to ABZ has a low capacity *in vitro* [33], moreover, considering that the treatment was performed on metacestodes tissues, the high concentrations (320, 160 and 80 μM) of ABZ was used. As controls, the metacestode slices were treated with the same medium with the same amounts of DMSO as in the drug-treated groups. Finally, glucose was added to a final concentration of 6 mM, and 0.2 mL samples were collected every 30 minutes up to 2 hours. Then, the samples were centrifuged at 10,000*g* for 10 minutes at 4°C. Aliquots of the supernatant were collected and subjected to lactic acid determination using the Lactic Assay Kit (Solarbio Biotech Company Ltd., Beijing, China). *A*_570_ values were read on an ELISA reader (Bio-Tek, Winooski, VT, USA), and lactic acid production was calculated by the standard equation acquired from the standard curve, which was established using the standard solution concentration (2.5, 1.25, 0.625, 0.3125, 0.15625, and 0.078 mM of lactic acid) as the X-axis and *A*_570_ as the Y-axis. The glucose concentration of the supernatant from each sample was determined by the glucose oxidase method. The amount of glucose consumption was calculated by the glucose concentrations of blank wells subtracting the remaining glucose in cell-plated wells. In the experiment, each drug concentration was performed in duplicate, and the experiments were repeated twice.

### Statistics analysis

Statistical analysis was performed with SPSS 19.0 software. The Kruskal–Wallis test followed by Dunn’s multiple-comparisons test was used for statistical comparisons. All data are shown as mean ± standard deviation (SD). The results were considered statistically significant at *p* < 0.05.

## Results

### 2-DG exhibits *in vitro* efficacy against *E*. *granulosus* protoscoleces

**Fig 1:**
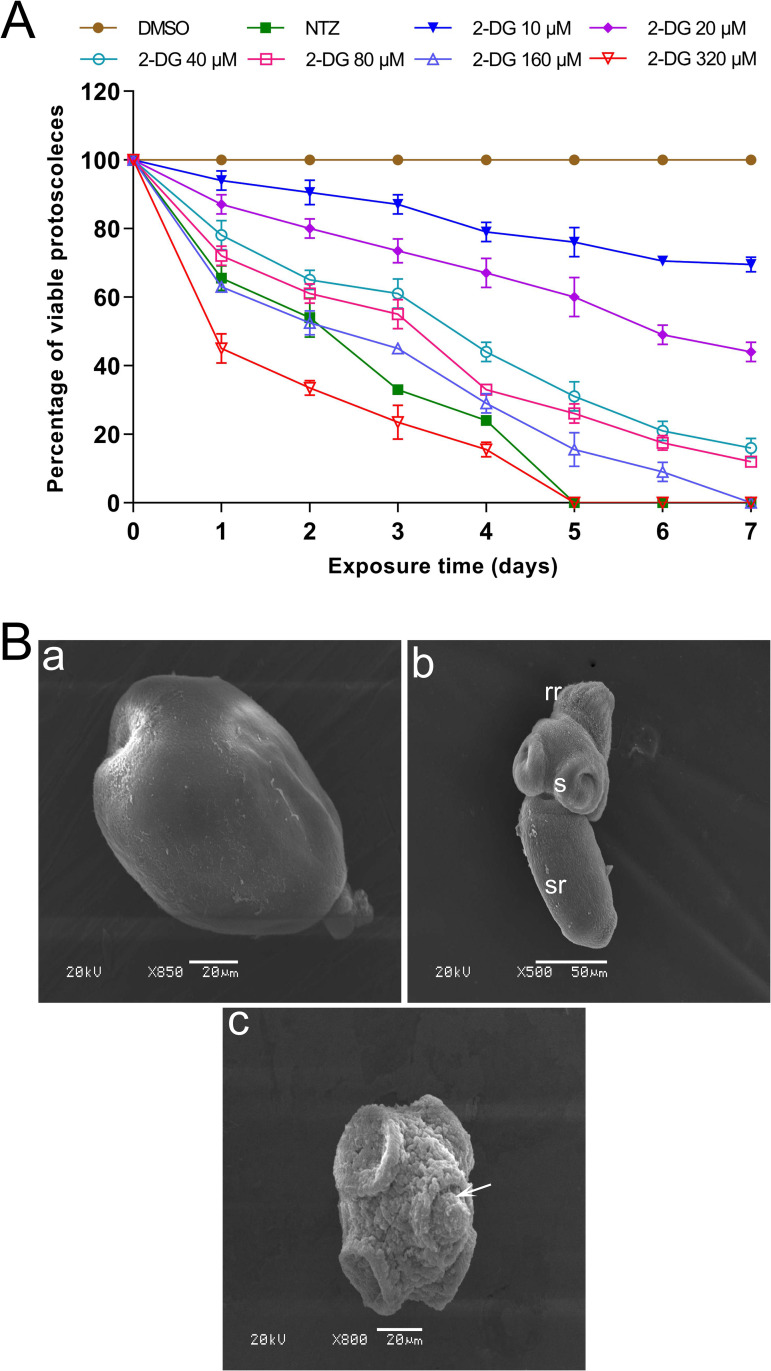
*In vitro* effect of 2-DG against *E*. *granulosus* protoscoleces. (A) Viability of protoscoleces incubated for 7 days with 40 μM NTZ or serial concentrations of 2-DG (10, 20, 40, 80, 160, and 320 μM). Protoscoleces incubated in a culture medium containing 0.2% DMSO were used as a control. (B) Scanning electron microscopy of protoscoleces after 5 days of incubation. (a) Invaginated control protoscoleces; (b) Evaginated control protoscoleces (rr, rostelar region; s, suckers; sr, soma region); (c) Protoscoleces incubated with 40 μM 2-DG. Protoscoleces showed marked contraction, shedding of microtriches and loss of hooks (arrow).

To investigate the *in vitro* effect of 2-DG on the viability on *E*. *granulosus* protoscoleces, the mortality of protoscoleces was assessed daily in response to serial concentrations of 2-DG (Fig 1A). At 320 μM and 160 μM concentrations, 100% protoscoleces were killed within 5 and 7 days, respectively. At 10, 20, 40, and 80 μM 2-DG, we found that 30% ± 2%, 56% ± 3%, 84% ± 3%, and 88% ± 2% of protoscoleces, respectively, were dead after 7-day exposure. S1 Fig shows the morphological changes of protoscoleces after 5-day treatment (without trypan blue staining). Compared with control protoscoleces, which exhibited normal morphology, the structure of the protoscoleces was markedly altered in the 2-DG–treated group in a dose-dependent manner, which was consistent with the viability assay. Moreover, SEM analysis confirmed the effects of 2-DG against protoscoleces on the ultrastructural level. After 5-day treatment with 40 μM 2-DG, the protoscoleces showed marked contraction, loss of hooks and rostellar disorganization, and shedding of microtriches in the scolex region (Fig 1B(c)). Treatment with 320 μM 2-DG led to a complete tegumental alteration and loss of the characteristic morphology (S2 Fig).

### 2-DG exhibits *in vitro* efficacy against *E*. *multilocularis* metacestodes

**Fig 2:**
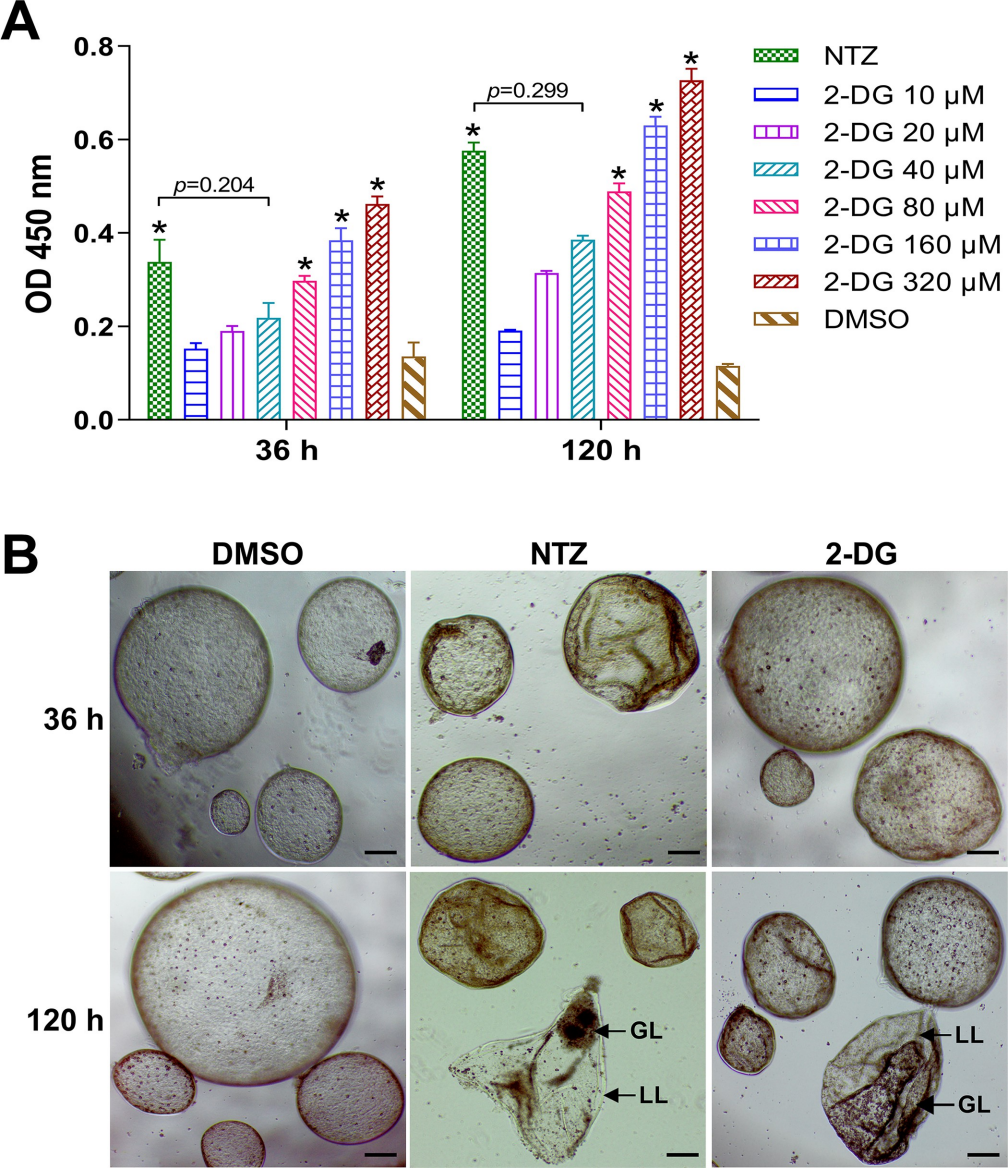
*In vitro* effects of 2-DG on *E*. *multilocularis* metacestodes. (A) *E*. *multilocularis* AP (*Em*AP) activities in the medium supernatant after 36 and 120 hours of treatment with 40 μM NTZ or various 2-DG concentrations (10, 20, 40, 80, 160, and 320 μM). Vesicles incubated in the culture medium containing DMSO served as controls. *p < 0.05 vs. DMSO control. (B) Microscopical damage to metacestodes treated with 40 μM NTZ or 40 μM 2-DG for 36 and 120 hours. Control vesicles exhibited no morphological changes, while drug-treated vesicles showed increased collapse and wrinkled cyst walls. LL, laminated layer; GL, germinal layer. Scale bar, 200 μm.

To investigate the *in vitro* effect of 2-DG on *E*. *multilocularis* metacestodes, the *Em*AP activities in culture supernatants were analyzed in response to a serial concentration of 2-DG. As shown in Fig 2A, the *Em*AP activities increased significantly and continuously with the increase in 2-DG concentration. After 36 hours of treatment, 80, 160, and 320 μM 2-DG led to a significant increase in AP activity, which indicated significant damage to metacestodes, compared with the DMSO control group. When the treatment time was extended to 120 hours, as expected, there was a higher release of the *Em*AP than at 36 hours. The results demonstrated that the effect of 2-DG on *E*. *multilocularis* metacestodes was time- and dose-dependent. The morphological alterations in metacestodes observed by optical microscope supported the *Em*AP activity results (Fig 2B). The loss of turgor of the vesicles was observed at 36 hours of 2-DG treatment, and obvious collapse and contraction of the metacestodes were detected at 120 hours of treatment.

### 2-DG and combined 2-DG/ABZ exhibit therapeutic efficacy against *E*. *multilocularis* infection in mice

**Fig 3:**
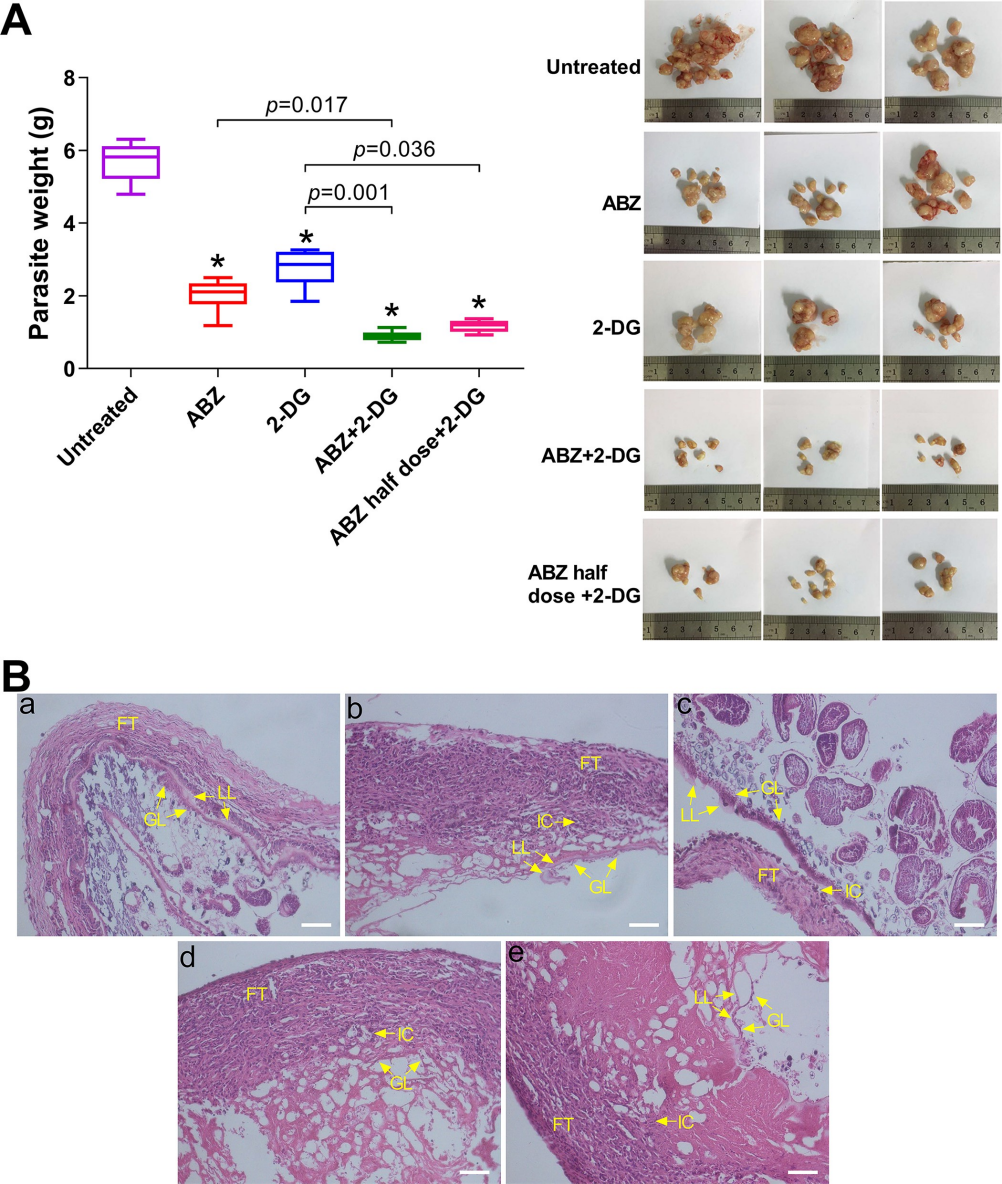
*In vivo* effect of 2-DG and its combination with ABZ in *E*. *multilocularis*–infected mice. (A) The weights and gross morphology of the metacestodes in untreated mice and mice treated with drugs for 6 weeks. *p < 0.05 vs. untreated mice. (B) Histopathological examination of *E*. *multilocularis* metacestodes from different treatment groups. a, mice infected with *E*. *multilocularis*; b, c, d, and e, infected mice treated with ABZ (200 mg/kg/day), 2-DG (500 mg/kg/day), ABZ + 2-DG (200 mg/kg/day + 500 mg/kg/day), and ABZ half-dose + 2-DG (100 mg/kg/day + 500 mg/kg/day), respectively. LL, laminated layer; GL, germinal layer; IC, inflammatory cell; FT, fibrous tissue. Scale bar, 50 μm.

To investigate the *in vivo* therapeutic effect of 2-DG, BALB/c mice were intraperitoneally infected with *E*. *multilocularis* metacestodes. The mice were treated 8 weeks later by oral administration of 2-DG (500 mg/kg/day), or ABZ (200 mg/kg/day), or the combination of ABZ and 2-DG (200 mg/kg/day + 500 mg/kg/day, or 100 mg/kg/day and 500 mg/kg/day) over a period of 6 weeks. After 6 weeks of treatment, in comparison to the untreated control, wet weights of metacestodes decreased in all of the treated groups. As indicated in Fig 3A, the treatment with 2-DG resulted in a significant reduction in wet weight by 53.1%, and ABZ treatment led to a 63.6% reduction in wet weight compared to untreated control. The combined therapy, that is, ABZ + 2-DG (1.071 ± 0.284 g) or ABZ half-dose + 2-DG (1.369 ± 0.231 g), caused significant reduction in parasite weights compared with the treatment with either of the drugs alone (2.005 ± 0.447 g for ABZ, and 2.763 ± 0.521 g for 2-DG treatments). There were no significant differences between the ABZ + 2-DG–treated group and the ABZ half-dose + 2-DG–treated group. No death and adverse effects occurred in the treated mice during the experiment. Histopathological examination indicated a typical structure of *E*. *multilocularis* metacestodes. The cysts were surrounded by host connective tissues, with clear germinal and laminated layers (a in Fig 3B). In the parasite tissue from ABZ–or 2-DG–treated mice, the laminated layers exhibited similar partially broken and fragmented structure (b and c in Fig 3B). The metacestodes from mice treated with ABZ + 2-DG or ABZ half-dose + 2-DG showed severe damage; the residual vesicles were encapsulated by proliferated and dense host fibrous tissue and were infiltrated with host’s inflammatory cells (d and e in Fig 3B). Both the germinal and laminated layers appeared to be partially dissolved and much thinner (~5-μm-thick) than those in untreated metacestodes (~15-μm-thick).

**Fig 4:**
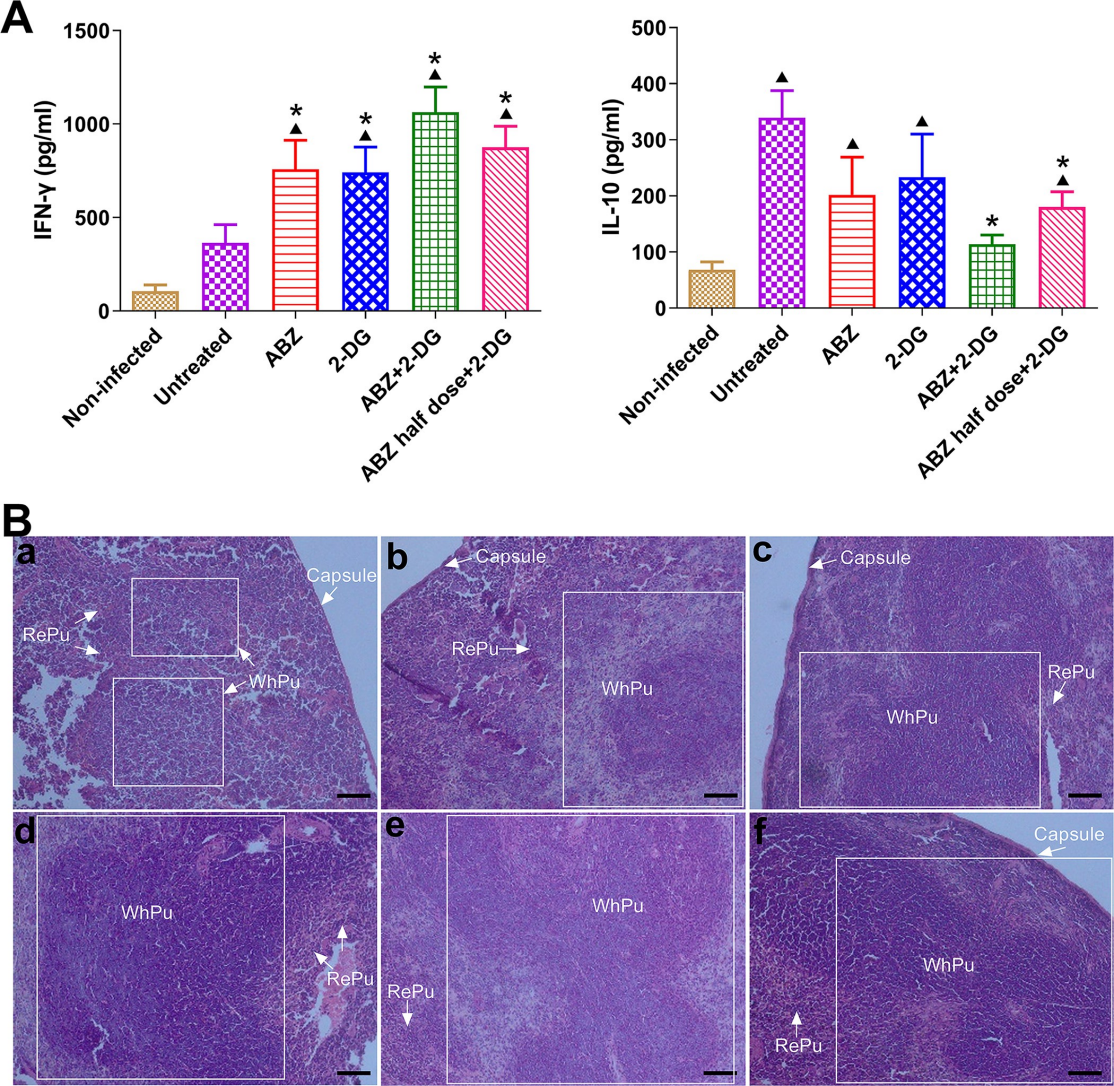
Cytokine profiles and histopathological examination. Serum concentrations of IFN-γ and IL-10 (A) were measured, and spleen tissues were observed (H&E staining) (B) from mice in different treatment groups. a, non-infected mice; b, mice infected with *E*. *multilocularis* metacestodes; c, d, e, and f, infected mice treated with ABZ, 2-DG, ABZ + 2-DG, and ABZ half-dose + 2-DG, respectively. RePu, the red pulp region in the spleen; WhPu, the white pulp region (lymphoid tissue containing lymphocyte production centers) in the spleen. *p < 0.05 vs. untreated mice, ▲*p* < 0.05 vs. non-infected mice. Scale bar, 50 μm.

To investigate the effects of the above treatments on Th1/Th2 cytokine profiles, the serum levels of IFN-γ and IL-10—representative Th1 and Th2 markers, respectively—were measured. As shown in Fig 4A, the levels of IFN-γ in all of the treatment groups were significantly elevated compared with those in the untreated mice. *E*. *multilocularis* infection resulted in a significant increase in IL-10 compared with non-infected mice; ABZ + 2-DG and ABZ half-dose + 2-DG were both able to significantly reduce the level of IL-10 compared with the untreated group. Although ABZ and 2-DG alone treatment also decreased the serum level of IL-10 in the infected mice, statistical significance was not achieved. The histopathological examination of spleens showed that the non-infected mice had normal splenic morphology (Fig 4B). After the infection with *E*. *multilocularis* metacestodes, the white pulp region (lymphoid tissue containing lymphocyte production centers) in the spleen presented proliferation. In mice treated with ABZ and 2-DG, the white pulp showed significant proliferation with irregular morphology. After treatment with the combination of ABZ and 2-DG, the spleen exhibited widespread proliferation of cells, with the loss of red/white pulp differentiation.

### Ultrastructural alterations of *E*. *multilocularis* metacestodes after 2-DG or combined 2-DG/ABZ treatment

**Fig 5:**
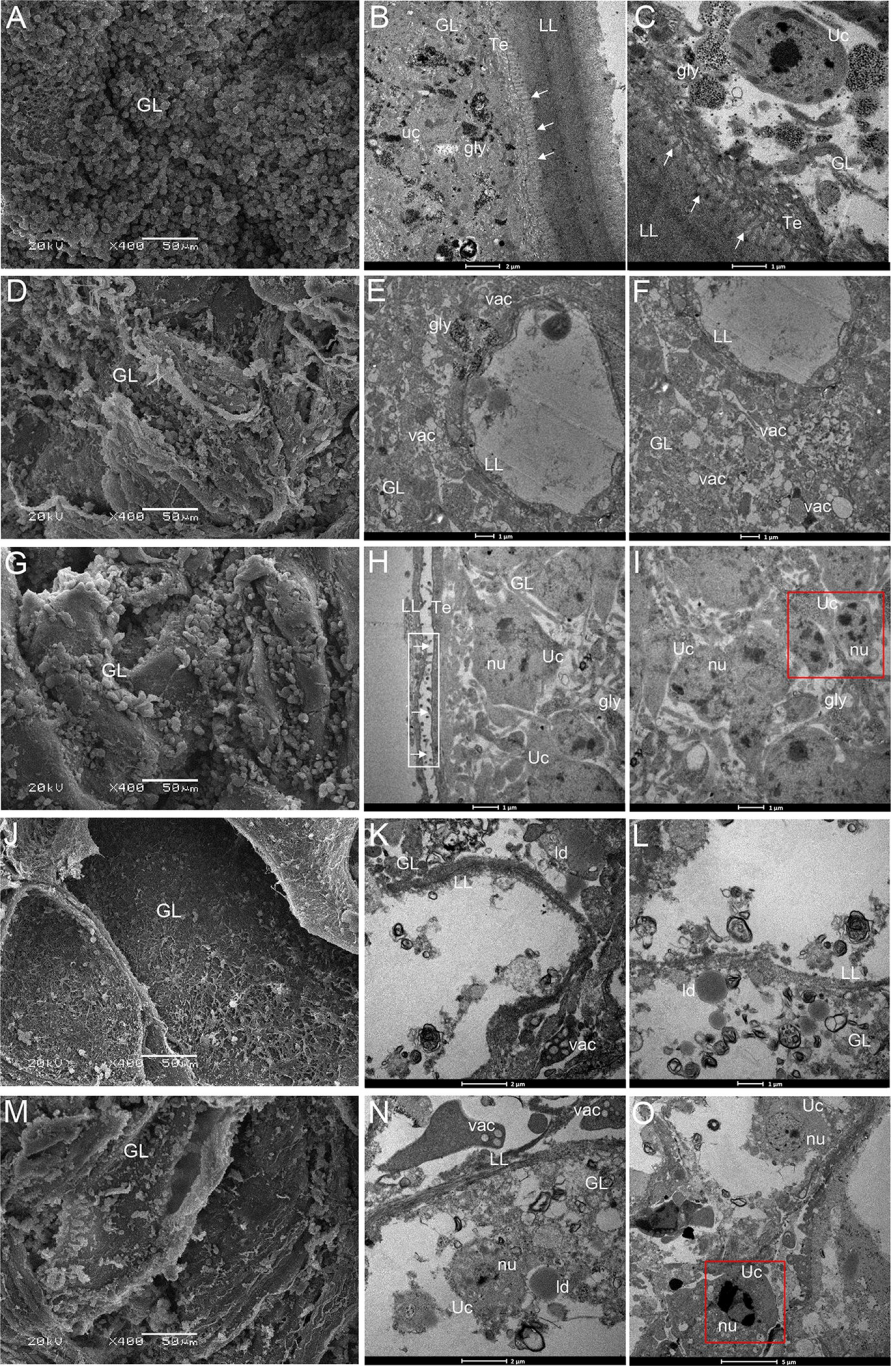
Ultrastructural alterations of metacestodes in different treatment groups. (A) Scanning electron microscopy (SEM) and transmission electron microscopy (TEM) (B and C) show metacestodes from untreated mice. The metacestode has a characteristic structure with an intact germinal layer surrounded by an acellular laminated layer. The arrows indicate microtriches originating from the tegument and protruding into the laminated layer. Note the large number and the length of microtriches. (D) SEM and TEM (E and F) show metacestodes from ABZ-treated mice. Note the complete absence of microtriches at the tegument and regression of the germinal layers. (G) SEM and TEM (H and I) show metacestodes from 2-DG–treated mice. Note the profound shortening and reduced number of the microtriches (marked by a white box and arrows) and a less dense appearance of the germinal layer tissue. Moreover, the characteristic features of apoptosis are visible in undifferentiated cells (marked by a red box). (J), (K), (L) and (M), (N), and (O) show metacestodes from mice treated with ABZ + 2-DG and ABZ half-dose + 2-DG. The metacestodes exhibited complete breakdown of the structural integrity of the vesicles; there was a complete loss of microtriches, and only residual laminated layers lacking any cellular parasites components were found in many areas. LL, laminated layer; GL, germinal layer; Teg, tegument; Uc, undifferentiated cells; gly, glycogen storage cells; vac, vacuolization of the tissue; nu, cell nucleus; ld, lipid drops.

The *in vivo* therapeutic effects of the drug were further evaluated by the ultrastructural manifestations using SEM and TEM. The parasites from untreated mice exhibited characteristic structure of *E*. *multilocularis* metacestodes (Fig 5A–5C), with an outer acellular laminated layer, tegument, and germinal layer. The tegument attached closely with the laminated layer, with a large number of microtriches protruding distinctly into the laminated layer. The inner germinal layer comprised densely packed parasite tissue that contained glycogen storage cells, connective tissue, and undifferentiated cells. The metacestodes from ABZ-treated mice appeared severely damaged; the microtriches appeared truncated or even absent, and there was regression of the germinal layer, which presented decreased number of viable cells compared with untreated metacestodes (Fig 5D–5F). 2-DG–treated parasite tissue also exhibited distinct changes, such as profound shortening and reduction of the microtriches, with a less dense appearance of the germinal layer tissue. Moreover, there were characteristic alterations of apoptosis in undifferentiated cells (Fig 5I), with chromatin condensation and margination, nuclear fragmentation, cell shrinkage, and cell blebbing. The metacestodes treated with ABZ + 2-DG or ABZ half-dose + 2-DG showed severe damage, with complete breakdown of the structural integrity of the vesicles. The microtriches were completely absent; in large areas, only residual laminated layers were observed, lacking any cellular parasites’ components. In addition, the tissue exhibited vacuolization and occurrence of lipid droplets, suggesting that the parasites were metabolically impaired during the treatment.

### Serum biochemical analysis of *E*. *multilocularis*–infected mice after 2-DG or combined 2-DG/ABZ treatment

**Fig 6:**
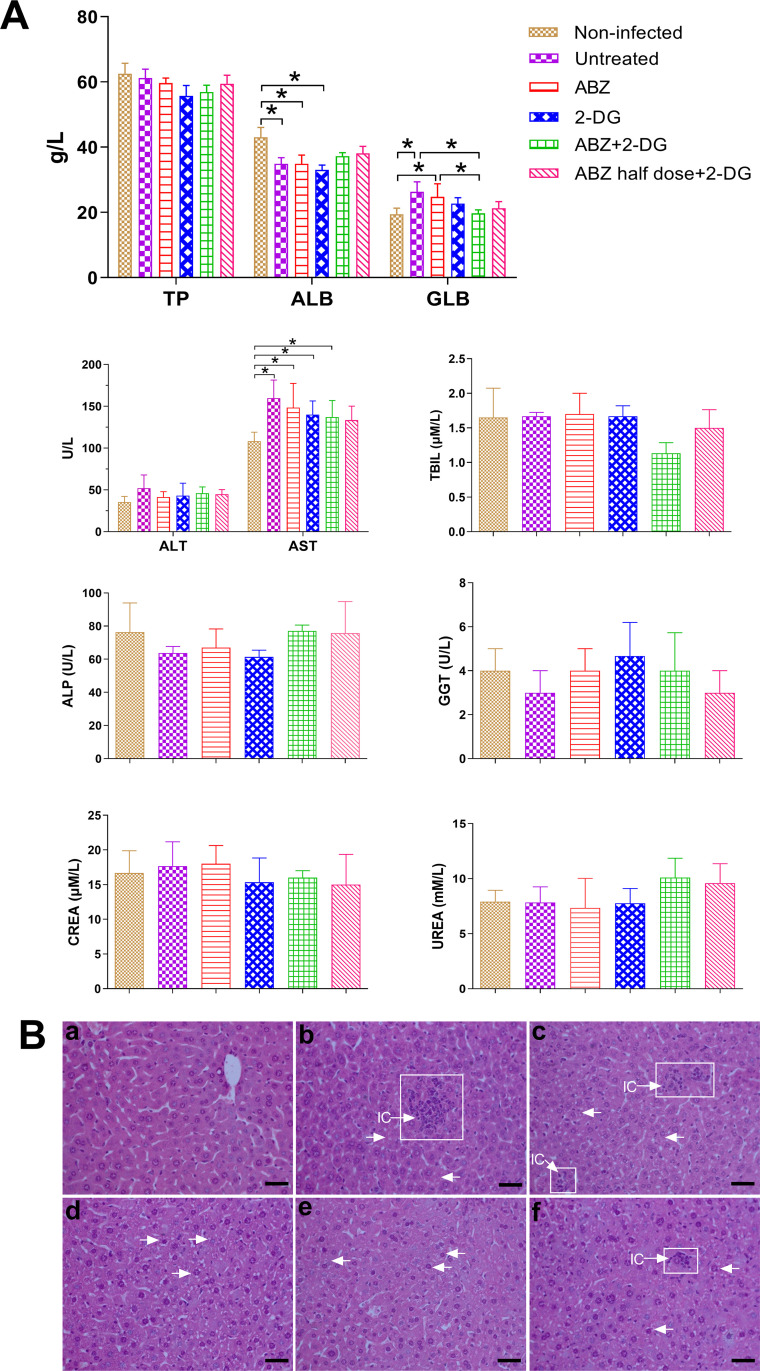
Serum biochemical analysis and histopathological examination in mice treated with ABZ, 2-DG, ABZ + 2-DG, or ABZ half-dose + 2-DG for 6 weeks. (A) The levels of total protein (TP), albumin (ALB), globulin (GLB), alanine aminotransferase (ALT), aspartate aminotransferase (AST), total bilirubin (TBIL), alkaline phosphatase (ALP), glutamyltranspeptidase (GGT), urea, and creatinine (CREA) in mice serum. *p < 0.05. (B) H&E images of liver tissues. Non-infected tissue (a), *E*. *multilocularis-*infected tissue (b), ABZ-treated tissue (c), 2-DG-treated tissue (d), ABZ + 2-DG–treated tissue (e), and ABZ half-dose + 2-DG–treated tissue (f) were visualized with a microscope. Focal necrosis is marked by a white box, and the white arrows indicate hepatocyte edema. IC, inflammatory cell. Scale bar, 50 μm.

To investigate the influence of drugs treatment on liver function, the level of biochemical parameters for hepatic and biliary functionality, including alanine aminotransferase (ALT), aspartate aminotransferase (AST), total protein (TP), albumin (ALB), globulin (GLB), alkaline phosphatase (ALP), glutamyltranspeptidase (GGT), and total bilirubin (TBIL), were measured in serum of the mice after 6 weeks of treatment (Fig 6A). At the same time, the common markers for kidney function, creatinine (CREA) and urea, were measured to investigated the influence of drugs treatment on glomerular filtration function. When liver damages appear, the serum levels of ALT, AST, ALP, GGT, TBIL will be increased; while those of TP and ALB will be decreased. When glomerular filtration function is damaged, urea and CREA will be increased. The significant decreases in ALB were observed in ABZ- and 2-DG–treated mice; in contrast, significant increases in GLB were observed in ABZ-treated mice. Nonetheless, no significant difference was found in TP levels between non-infected mice and drugs-treated mice. The AST levels in untreated mice, as well as ABZ, 2-DG, and ABZ + 2-DG treated mice were significantly higher than those in non-infected mice. Although the AST levels of mice in the drugs-treated groups were all lower than those in the untreated mice, the decreases showed no statistical significance. Other measured liver and kidney function indexes after treatment indicated no significant changes compared with the non-infected or untreated mice. The further histopathological examination of the liver from the drugs-treated mice was compatible with the changes observed in the biochemical parameters (Fig 6B). As shown in (b) of Fig 6B, the infection by *E*. *multilocularis* metacestodes resulted in distinct focal necrosis, cell swelling, and the infiltration of inflammatory cells in the liver, compared with non-infected liver tissue (a). The livers from the drugs-treated mice exhibited inflammatory cells infiltration, with mild cellular swelling of hepatocyte (Fig 6B (c–f)).

### 2-DG or combined 2-DG/ABZ treatment induces apoptosis of *E*. *multilocularis* metacestodes in mice

**Fig 7:**
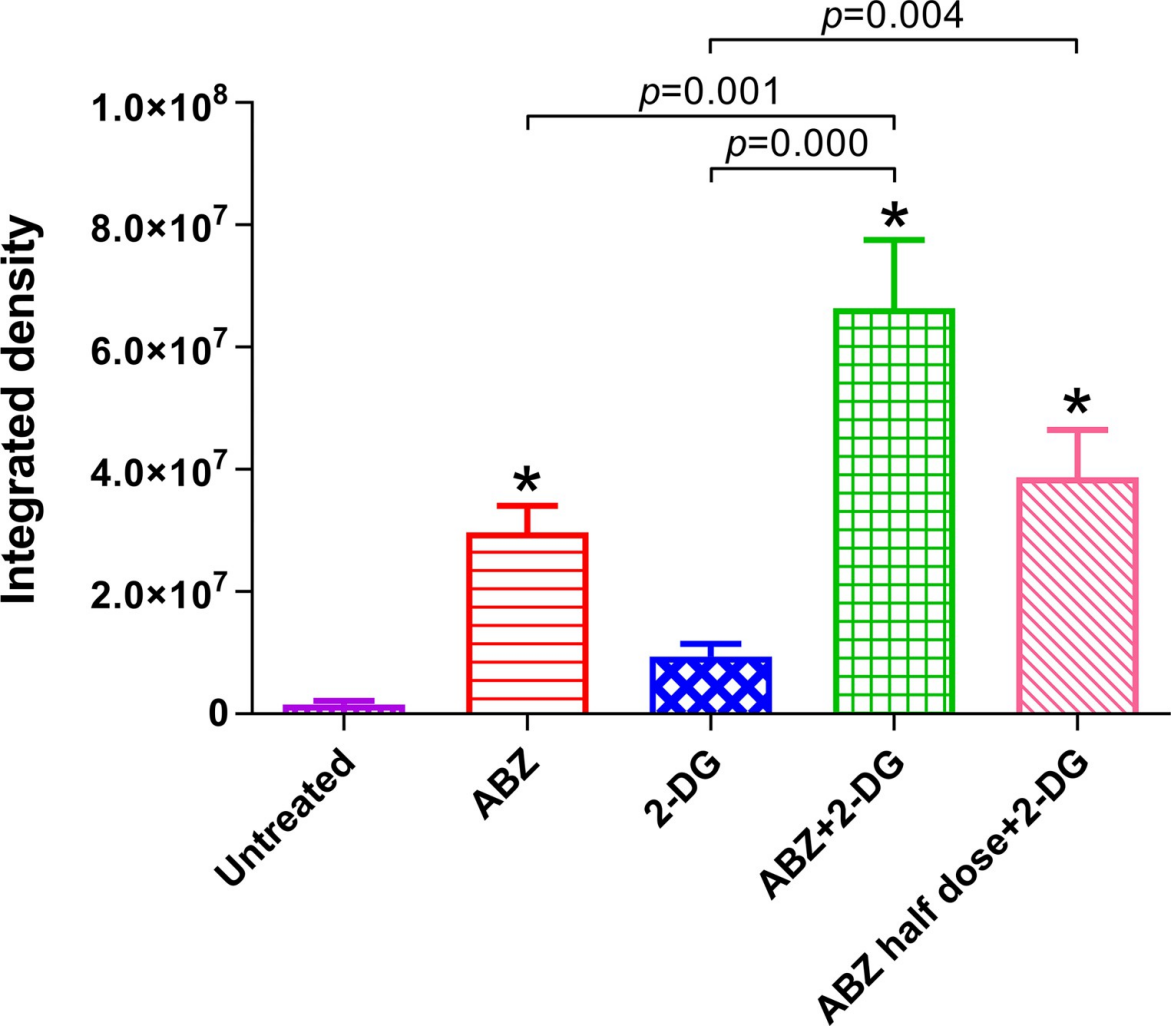
Analysis of apoptosis in *E*. *multilocularis* metacestodes. The TUNEL staining was performed in metacestodes from untreated mice, mice treated with ABZ (200 mg/kg/day), 2-DG (500 mg/kg/day), ABZ + 2-DG (200 mg/kg/day + 500 mg/kg/day), or ABZ half-dose + 2-DG (100 mg/kg/day + 500 mg/kg/day). Apoptosis was evaluated by the integrated density of green fluorescence in metacestodes using ImageJ software. *p < 0.05 vs. untreated mice.

To investigate the effects of 2-DG and combined 2-DG/ABZ on apoptosis of *E*. *multilocularis* metacestodes in mice, TUNEL staining analysis was performed (Figs 7 and S3.). The results demonstrated that the integral optical density values of FITC fluorescence in the metacestodes treated with ABZ + 2-DG and ABZ half-dose + 2-DG were significantly higher than those in the untreated and 2-DG alone–treated metacestodes. Although the integral optical density value was higher in 2-DG alone–treated metacestodes compared with untreated metacestodes, the increase did not reach statistical significance.

### Effects of 2-DG or combined 2-DG/ABZ on glucose intake and lactate production of *E*. *multilocularis* metacestodes

**Fig 8:**
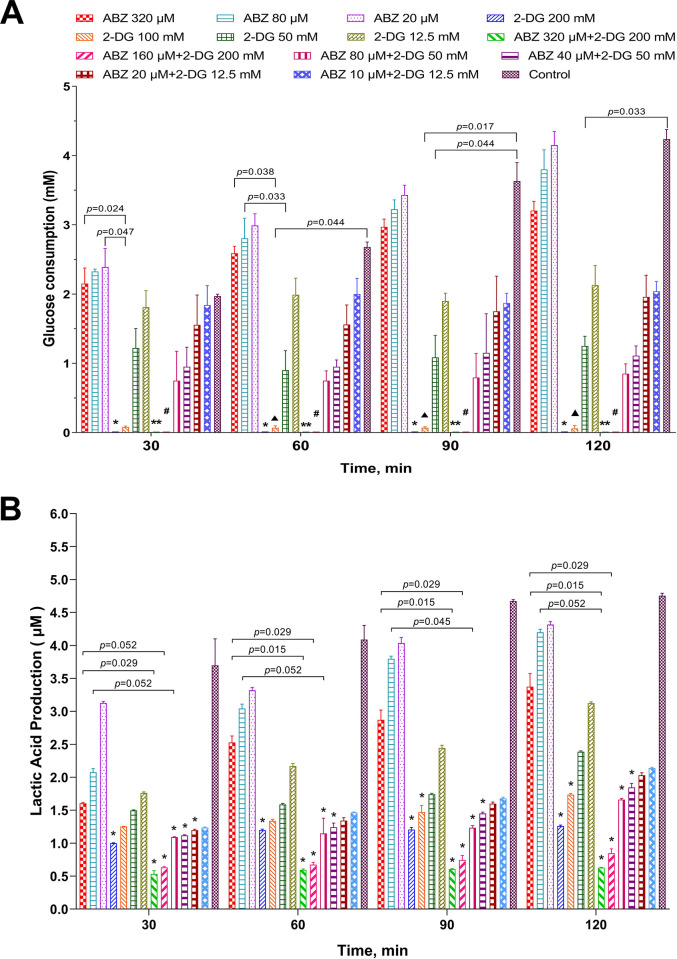
Effects of ABZ, 2-DG, ABZ + 2-DG, or ABZ half-dose + 2-DG on glucose metabolism of *E*. *multilocularis* metacestodes. Various concentrations of ABZ, 2-DG, or combined ABZ/2-DG were preincubated with 200 μg metacestodes slices for 30 minutes at 37°C, and then the slices were incubated in the presence of 6 mM glucose. (A) Glucose consumption was determined for the times indicated. *p < 0.05, 2-DG 200 mM–treated group vs. control group, ABZ 320 μM, ABZ 80 μM, or ABZ 20 μM–treated group. **p < 0.05, ABZ 320 μM + 2-DG 200 mM vs. control group, ABZ 320 μM, ABZ 80 μM, or ABZ 20 μM–treated group. **#***p* < 0.05, ABZ 160 μM + 2-DG 200 mM vs. control group, ABZ 320 μM, ABZ 80 μM, or ABZ 20 μM–treated group. ▲*p* < 0.05, 2-DG 100 mM–treated group vs. control group or ABZ 80 μM–treated group. (B) Lactate production in culture medium for the times indicated. *p < 0.05 vs. control group.

To investigate the effects of 2-DG and combined 2-DG/ABZ on glucose metabolism, the glucose consumption and lactate production in metacestodes after treatment with 2-DG, ABZ, and their combination were measured (Fig 8). Both glucose intake and lactate production in metacestodes decreased significantly after the treatment with 200 mM or 100 mM 2-DG compared with control, while 50 mM and 12.5 mM 2-DG did not result in statistically significant decreases (*p*>0.05). The results revealed that 2-DG exerted a time- and dose-dependent inhibitory effect on glucose consumption and lactate production of metacestodes. In the combination treatment group, ABZ 320 μM + 2-DG 200 mM and ABZ 160 μM + 2-DG 200 mM both significantly decreased glucose consumption and lactate production in metacestodes compared with the control group. The decreases after the combination treatment showed no significant difference compared with those after the treatment with 2-DG alone; in contrast, a significant difference was observed relative to the results after the treatment with ABZ alone.

## Discussion

Previous studies revealed that the larval stages of both *E*. *granulosus* and *E*. *multilocularis* mainly rely on glycolysis for energy production and glycolysis intermediates for other metabolic processes [34, 35]. On the other hand, humans are much less sensitive to the inhibition of glycolysis by possessing multiple hexokinase (the first rate-limiting enzyme of glycolysis) isoforms (HK-1, HK-2, HK-3 and HK-4), a fully functional Krebs cycle and the ability to use alternative energy sources (e.g., amino acids or fatty acids). Therefore, the inhibition of glycolysis of metacestodes may be an effective anti-parasitic strategy against *Echinococcus*. In our previous study, a series of glycolytic inhibitors exhibited effective activity against both *E*. *granulosus* and *E*. *multilocularis* [19, 20]. Among those glycolytic inhibitors, 3-bromopyruvate as an inhibitor of hexokinase showed the highest anti-*Echinococcus* activity, which suggests that it may be a promising anti-parasitic agent against *Echinococcus* to block glycolysis metabolism by inhibiting the activity of hexokinase. Therefore, in this study, we investigated the anti-*Echinococcus* effect of 2-DG, which is a glucose analog and can act as a competitive inhibitor of hexokinase [36–38].

2-DG exhibited the *in vitro* efficacy against *E*. *granulosus* protoscoleces in a time- and dose-dependent manner. Protoscoleces exposed to 2-DG showed morphological alterations such as soma contraction, rostellar disorganization, and tegument destruction (Fig 1). The efficacy of 2-DG was also demonstrated *in vitro* on *E*. *multilocularis* metacestodes; namely, 2-DG resulted in structural damage to metacestodes and loss of viability (Fig 2). *E*. *multilocularis* metacestodes exhibit properties of asexual, unlimited and infltrative proliferation in humans. The germinal layer of metacestode can differentiate into various tissues, including brood capsules and protoscoleces, indicating the its significance in parasite survival. Our *in vitro* efficacy study was performed in protoscoleces of *E*. *granulosus* and metacestodes of *E*. *multilocularis*, respectively, demonstrating that 2-DG has potent effects against the larval stage of both *E*. *granulosus* and *E*. *multilocularis*.

In order to investigate the *in vivo* therapeutic effect of 2-DG, BALB/c mice were intraperitoneally infected with *E*. *multilocularis* metacestodes. Ideally, experiment should be performed in mice infected by oral inoculation of *E*. *multilocularis* eggs. However, because of the condition limitations, this mice model was not available in our experiment. However, in these two infection models, either primary model (larval *E*. *multilocularis* lives in liver) or secondary model (larval *E*. *multilocularis* lives in peritoneal cavity), metacestodes proliferate in an extremely-low-oxygen environment. Therefore, this secondary AE model is suitable for our investigation. The *in vitro* effect of 2-DG was further confirmed in the mice model of AE. Namely, 2-DG treatment (500 mg/kg/day) resulted in a significant reduction in parasite burden (by 53.1%) compared with the untreated mice (Fig 3A). Moreover, compared to the standard ABZ treatment (200 mg/kg applied orally on a daily basis) [10], the *in vivo* effect of 2-DG was comparable to that observed with ABZ after 6 weeks of administration (63.6% reduction in parasite weights), indicating the effective therapeutic activity of 2-DG for the treatment of *E*. *multilocularis* infection. The development of combination treatments is an important and useful strategy for the enhancement of effectiveness, shortening of treatment course, reduction of toxicity, and delay of drug resistance [39, 40]. Our results showed that the combined administration of 2-DG and ABZ exhibited a significant anti-AE effect compared with untreated mice. Notably, the combined treatment, either 2-DG (500 mg/kg/day) + ABZ (200 mg/kg/day) or 2-DG (500 mg/kg/day) + half-dose of ABZ (100 mg/kg/day), achieved an even more potent effect on the reduction of cysts’ weight than the administration of 2-DG or ABZ alone, indicating the synergistic effect elicited by the combination.

The histological examination of metacestodes verified the efficacy of 2-DG and the combined treatment (Fig 3B). The structural damage occurred mainly in the laminated layers and germinal layers. In addition, the increased infiltration of inflammatory cells in host fibrous tissue into residual vesicles suggests that 2-DG and the combined treatment have elicited the host immune response to inhibit the cyst growth. The spleen is a lymphatic organ and plays an important role in storing and releasing certain types of immune cells that mediate tissue inflammation. The spleen from the infected mice exhibited proliferation of the white pulp region, indicating that the infection by *E*. *multilocularis* metacestodes had stimulated the proliferation of lymphocytes in the host. 2-DG treatment, especially the combination of ABZ and 2-DG (Fig 4B), induced significant and widespread proliferation of cells in the white pulp and resulted in the loss of red/white pulp differentiation, thereby indicating the effective stimulation and activation of lymphocytes to fight against parasites.

Previous studies have shown that T helper cell (Th1)-type immune response might be important for the reduction or abrogation of metacestodes proliferation and in favor of protection, while Th2-type immune response is associated with the chronic and progressive course of the disease [41, 42]. IFN-γ favors Th1-type immune response and can inhibit metacestode growth and dissemination in *E*. *multilocularis*-infected mice [43], thereby stopping the disease progression in AE patients [44]. In contrast, IL-10, the immunological hallmark of patients with progressive forms of AE, inhibits cytokine production by Th1 cells, thereby leading to Th2-type immune response. During *E*. *multilocularis* infection, there is an imbalance between Th1-type and Th2-type immune responses, which is indicated by a shift from the initial Th1-oriented response to a more dominant Th2-oriented response, which is conducive to the immune evasion and growth of metacestodes [45]. 2-DG treatment, combination treatment with ABZ + 2-DG, and ABZ half-dose + 2-DG significantly elevated the serum levels of IFN-γ in mice compared with untreated mice; in contrast, these treatments decreased the serum levels of IL-10 in mice (Fig 4A). Therefore, the high levels of IFN-γ after the treatment indicated that 2-DG and the combination of ABZ and 2-DG had enhanced host immunity to inhibit the metacestodes growth. At the same time, the suppressed level of IL-10 induced by the treatment resulted in a shift from a more dominant Th2-type immune response to Th1-type immune response.

The *in vivo* anti-*Echinococcus* effects of 2-DG and the combination of ABZ and 2-DG were confirmed at the ultrastructural level by SEM and TEM. The metacestodes from 2-DG–treated mice exhibited profound shortening and reduced number of microtriches, and apoptosis in undifferentiated cells (Fig 5). Similar to previous reports [8, 46], ABZ-treated metacestodes in our experiment exhibited truncated or even absent microtriches (Fig 5). The microtriches are directly associated with nutrient uptake of metacestodes, and their alteration probably contributes to the loss of cyst viability [47]. The combination of ABZ and 2-DG induced more serious damage to the structure of metacestodes than the treatment with 2-DG or ABZ alone. In addition, the TUNEL staining analysis showed that combined 2-DG/ABZ induced apoptosis of the cells of metacestodes. The combination of 2-DG and ABZ significantly promoted apoptosis of metacestodes, thereby resulting in significant increases in integrated optical density, which indicates the synergistic effect elicited by the combination (Fig 7). There is evidence indicating that ABZ (or its metabolites) can bind to the parasite’s beta-tubulin and inhibit the assembly of microtubules *in vivo* [48, 49], resulting in the impairment of the cellular cytoskeleton. Associated with this effect, the microtubule-mediated, absorptive/secretory vesicular transport of helminth is interrupted, leading to the inhibition of glucose uptake and depletion of glycogen stores and consequently energy reduction of the parasite [50–52]. 2-DG is a stable glucose analog and competitively inhibits glucose uptake because they are both transferred by the glucose transporters [53]. After entering the cells, 2-DG is phosphorylated to 2-deoxyglucose 6-phosphate (2-DG-P) by hexokinase. However, this product is a poor substrate of glucose 6-phosphatase; thus, unlike glucose 6-phosphate (phosphorylated product of glucose by hexokinase), 2-DG-P cannot be further metabolized by phosphohexose isomerase, which converts glucose 6-phosphate to fructose-6-phosphate. Consequently, 2-DG-P is trapped and accumulated in the cell, interfering with glycolysis to deplete cellular energy by inhibiting the glycolytic enzyme hexokinase [36–38], eventually leading to a reduction in intracellular energy levels, blockage of cell cycle progression, inhibited cell growth, and even cell death [54–56]. Our results showed that 2-DG inhibited the glycolysis level; moreover, it significantly decreased cellular uptake of glucose and lactate production in *E*. *multilocularis* metacestodes. In addition, the combination of ABZ and 2-DG inhibited the glycolysis more effectively than the administration of ABZ alone (Fig 8). Given that 2-DG and ABZ have different cellular targets in term of biochemical actions, our study could imply that during the combination treatment of ABZ and 2-DG, the significantly enhanced inhibition of glycolysis was accompanied with the simultaneous impairment of cellular microtubular structures by ABZ and the interference of glycolysis by 2-DG; moreover, the significantly enhanced apoptosis in metacestodes might be responsible for the greater effectiveness against *E*. *multilocularis*. Nonetheless, further experiments are required to investigate the underlying mechanisms of increased effectiveness of the combination therapy.

2-DG has been proven as a promising treatment agent for many cancers [23–25] and an antiviral drug against human papillomavirus [22]. In addition, at present, at least one phase II clinical trial investigating the efficacy of 2-DG in COVID-19 (an infectious disease caused by Coronavirus 2, with a severe acute respiratory syndrome) patients is underway (CTRI/2020/06/025,664; Cochrane Central register of Controlled Trails) [57]. Previous phase I and phase II clinical trials in patients have shown that the administration of 2-DG alone or the combined treatment of 2-DG and other anticancer drugs/radiotherapy is safe and well tolerated by patients [24, 58–60]. Furthermore, in our *in vivo* experiments on murine AE, none of the mice experienced adverse effects throughout the treatments, and no significant differences in the biochemical parameters of the liver and kidney function (TP, ALT, AST, TBIL, ALP, GGT, urea, and CREA) were found (Fig 6). These results demonstrated that the administration of 2-DG alone or the combined treatment of ABZ and 2-DG did not induce significant toxicity in mice at the effective dose against *E*. *multilocularis* metacestodes, confirming the safety of 2-DG and the combined treatment for AE. Therefore, the efficacy and safety of 2-DG and the combination treatment of ABZ and 2-DG open the possibility for clinical application against echinococcosis in the future.

In conclusion, our results demonstrated that 2-DG exhibited effective *in vitro* activity and *in vivo* therapeutic activity against *Echinococcus*. Furthermore, the combined treatment of 2-DG and ABZ exhibited higher treatment efficiency than the treatment with 2-DG or ABZ alone. 2-DG is a promising anti-*Echinococcus* drug, and its combination with ABZ may provide a new strategy for the treatment of echinococcosis in humans in the future.

## Supporting information

S1 Fig The effects of NTZ or 2-DG on the morphology and structural integrity of protoscoleces. Light microscopy of protoscoleces incubated for 5 days with 40 μM NTZ and various 2-DG concentrations (10, 20, 40, 80, 160, and 320 μM). Protoscoleces incubated in culture medium containing DMSO served as a control. h, hooks; cc, calcareus corpuscles. Arrowhead points towards vesiculated protoscoleces. Scale bar, 100 μm.
(PDF)

S2 Fig Scanning electron microscopy of protoscoleces incubated for 5 days with 2-DG. a, Evaginated control protoscoleces (rr, rostelar region; s, suckers; sr, soma region); b, Contraction of the soma region and shedding of microtriches in the scolex region were observed in protoscoleces incubated with 10 μM 2-DG; c, Vesiculation, shedding of microtriches and tegument changes appeared in protoscoleces incubated with 20 μM 2-DG; d, Protoscoleces incubated with 80 μM 2-DG showed that the soma region appeared wrinkled, shedding of microtriches, rostellar disorganization, and loss of hooks (arrowhead); e, Loss of microtriches and hooks (arrowhead), and information of blebs (arrow) were observed in protoscoleces incubated with 160 μM 2-DG; f, Protoscoleces incubated with 320 μM 2-DG showed complete tegumental alteration and loss of the characteristic morphology, loss of hooks and absence of microtriches.
(PDF)

S3 Fig The demonstration of apoptosis in *E*. *multilocularis* metacestodes from mice treated with ABZ, 2-DG, ABZ + 2-DG or ABZ-half dose + 2-DG for 6 weeks. The slices were stained with TUNEL (green) and DAPI (blue). GL, germinal layer. FT, fibrous tissue; PSC, protoscoleces. Scale bar, 50 μm.
(PDF)
