# Supplementary material for: 2-Deoxy-D-glucose and combined 2-Deoxy-D-glucose/albendazole exhibit therapeutic efficacy against Echinococcus granulosus protoscoleces and experimental alveolar echinococcosis
Source: PLoS Negl Trop Dis. 2022 Jul 18;16(7):e0010618. doi: 10.1371/journal.pntd.0010618 (PMC9333451; doi:10.1371/journal.pntd.0010618)
Supplement: S1 Fig — Light microscopy of protoscoleces incubated for 5 days with 40 μM NTZ and various 2-DG concentrations (10, 20, 40, 80, 160, and 320 μM). Protoscoleces incubated in culture medium containing DMSO served as a control. h, hooks; cc, calcareus corpuscles. Arrowhead points towards vesiculated protoscoleces. Scale bar, 100 μm. (PDF) [file pntd.0010618.s001.pdf]

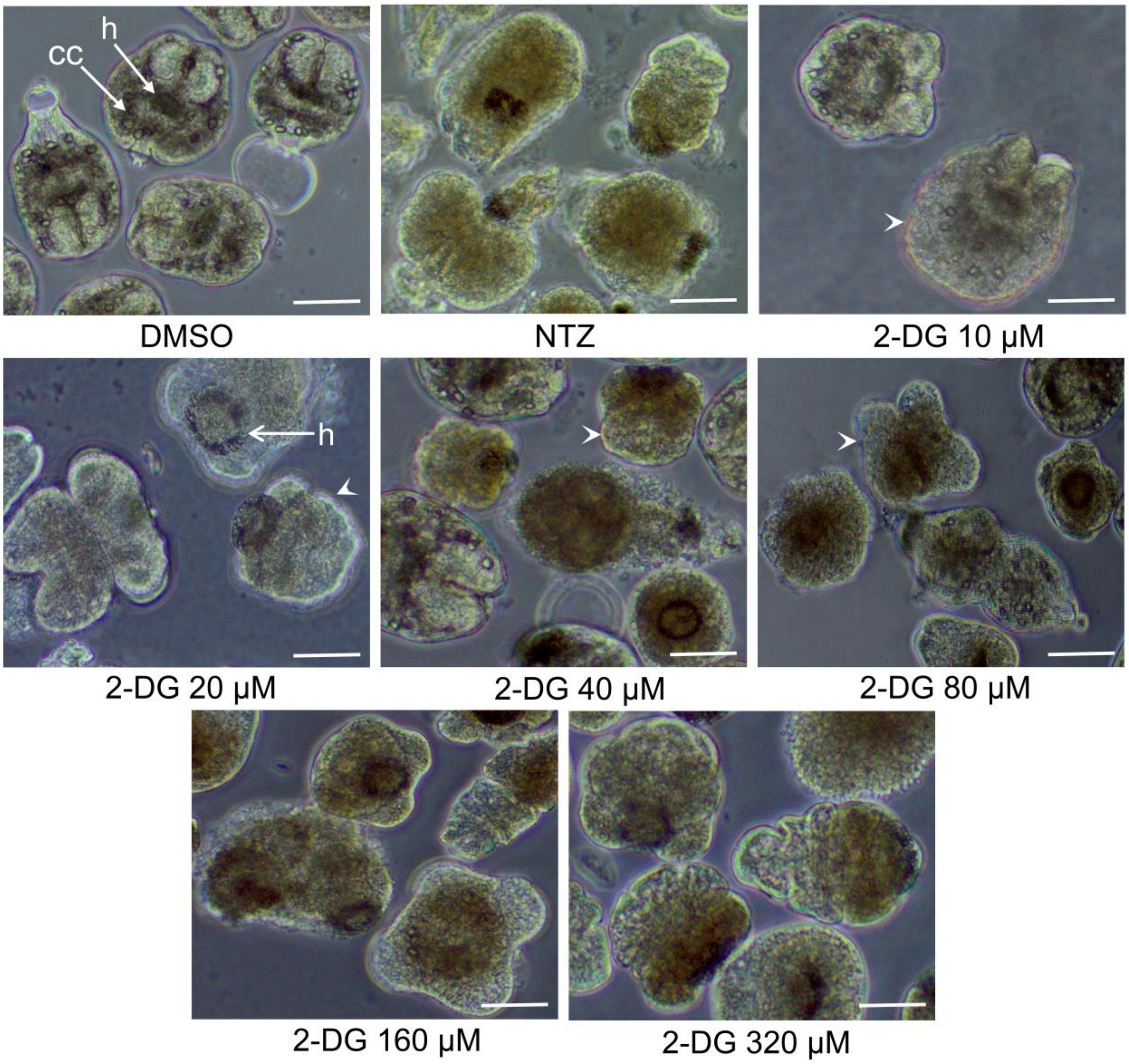

S1 Fig. The effects of NTZ or 2-DG on the morphology and structural integrity of protoscoleces. Light microscopy of protoscoleces incubated for 5 days with 40 µM NTZ and various 2-DG concentrations (10, 20, 40, 80, 160, and 320 µM). Protoscoleces incubated in culture medium containing DMSO served as a control. h, hooks; cc, calcareus corpuscles. Arrowhead points towards vesiculated protoscoleces. Scale bar, 100 µm.
