# Supplementary material for: 2-Deoxy-D-glucose and combined 2-Deoxy-D-glucose/albendazole exhibit therapeutic efficacy against Echinococcus granulosus protoscoleces and experimental alveolar echinococcosis
Source: PLoS Negl Trop Dis. 2022 Jul 18;16(7):e0010618. doi: 10.1371/journal.pntd.0010618 (PMC9333451; doi:10.1371/journal.pntd.0010618)
Supplement: S2 Fig — a, Evaginated control protoscoleces (rr, rostelar region; s, suckers; sr, soma region); b, Contraction of the soma region and shedding of microtriches in the scolex region were observed in protoscoleces incubated with 10 μM 2-DG; c, Vesiculation, shedding of microtriches and tegument changes appeared in protoscoleces incubated with 20 μM 2-DG; d, Protoscoleces incubated with 80 μM 2-DG showed that the soma region appeared wrinkled, shedding of microtriches, rostellar disorganization, and loss of hooks (arrowhead); e, Loss of microtriches and hooks (arrowhead), and information of blebs (arrow) were observed in protoscoleces incubated with 160 μM 2-DG; f, Protoscoleces incubated with 320 μM 2-DG showed complete tegumental alteration and loss of the characteristic morphology, loss of hooks and absence of microtriches. (PDF) [file pntd.0010618.s002.pdf]

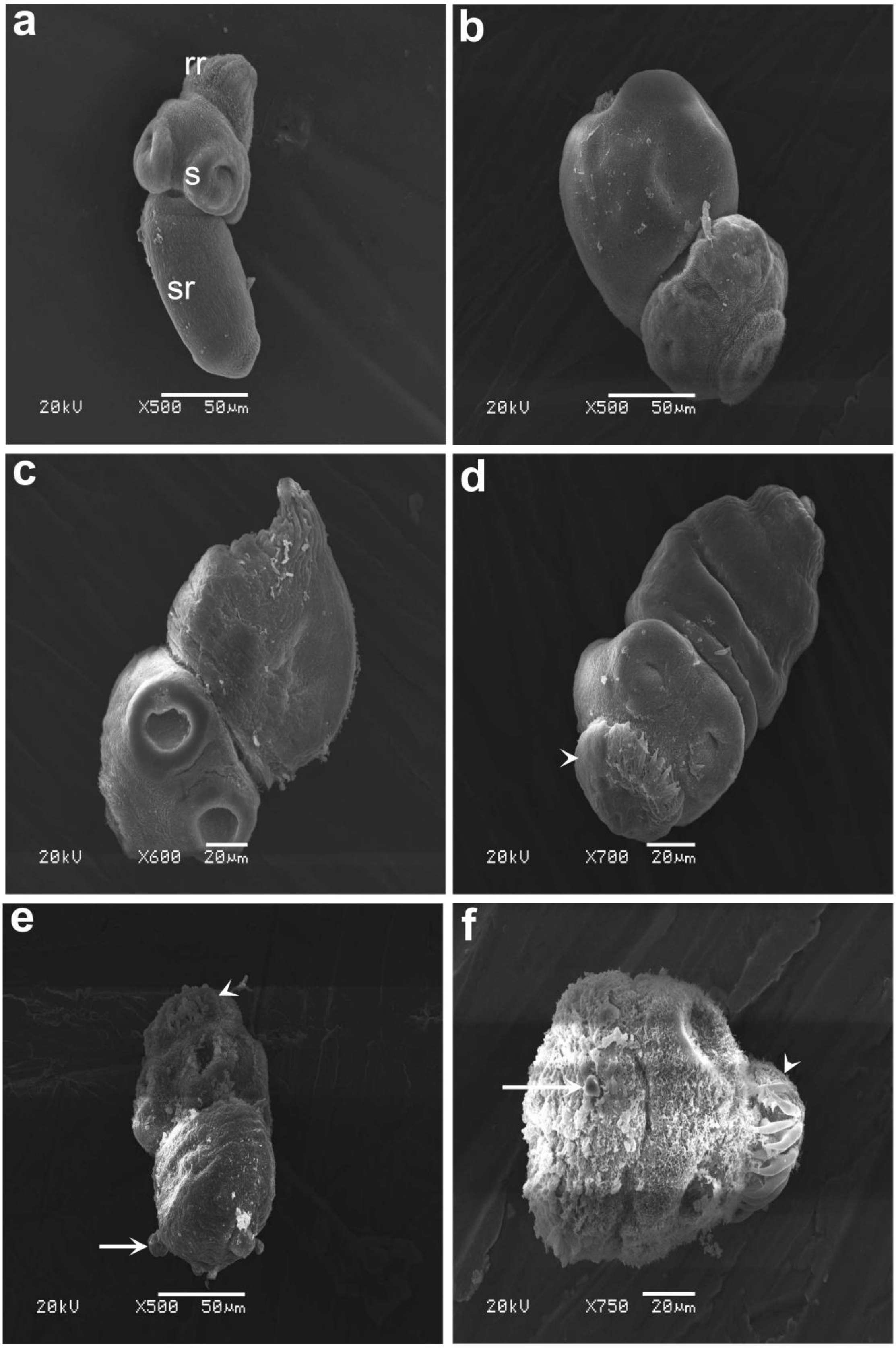

S2 Fig. Scanning electron microscopy of protoscoleces incubated for 5 days with

2-DG. a, Evaginated control protoscoleces (rr, rostelar region; s, suckers; sr, soma region); b, Contraction of the soma region and shedding of microtriches in the scolex region were observed in protoscoleces incubated with 10 μM 2-DG; c, Vesiculation, shedding of microtriches and tegument changes appeared in protoscoleces incubated with 20 μM 2-DG; d, Protoscoleces incubated with 80 μM 2-DG showed that the soma region appeared wrinkled, shedding of microtriches, rostellar disorganization, and loss of hooks (arrowhead); e, Loss of microtriches and hooks (arrowhead), and information of blebs (arrow) were observed in protoscoleces incubated with 160 μM 2-DG; f, Protoscoleces incubated with 320 μM 2-DG showed complete tegumental alteration and loss of the characteristic morphology, loss of hooks and absence of microtriches.
