# Supplementary material for: 2-Deoxy-D-glucose and combined 2-Deoxy-D-glucose/albendazole exhibit therapeutic efficacy against Echinococcus granulosus protoscoleces and experimental alveolar echinococcosis
Source: PLoS Negl Trop Dis. 2022 Jul 18;16(7):e0010618. doi: 10.1371/journal.pntd.0010618 (PMC9333451; doi:10.1371/journal.pntd.0010618)
Supplement: S3 Fig — The slices were stained with TUNEL (green) and DAPI (blue). GL, germinal layer. FT, fibrous tissue; PSC, protoscoleces. Scale bar, 50 μm. (PDF) [file pntd.0010618.s003.pdf]

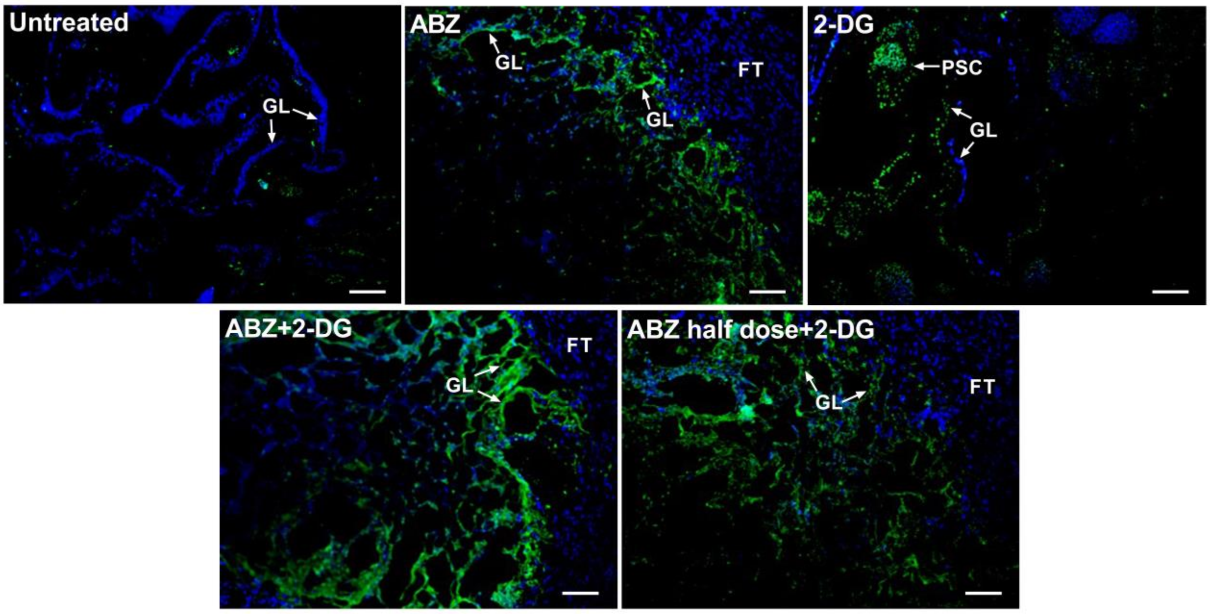

S3 Fig. The demonstration of apoptosis in *E. multilocularis* metacestodes from mice treated with ABZ, 2-DG, ABZ + 2-DG or ABZ-half dose + 2-DG for 6 weeks. The slices were stained with TUNEL (green) and DAPI (blue). GL, germinal layer. FT, fibrous tissue; PSC, protoscoleces. Scale bar, 50 μm.
